# Design of a Novel Delivery Efficiency Feedback System for Biphasic Dissolving Microarray Patches Based on Poly(Lactic Acid) and Moisture‐Indicating Silica

**DOI:** 10.1002/adhm.202304082

**Published:** 2024-03-19

**Authors:** Huanhuan Li, Qonita Kurnia Anjani, Aaron R. J. Hutton, Juan Luis Paris, Natalia Moreno‐Castellanos, Achmad Himawan, Eneko Larrañeta, Ryan F. Donnelly

**Affiliations:** ^1^ School of Pharmacy Queen's University Belfast Belfast BT9 7BL UK; ^2^ Instituto de Investigación Biomédica de Málaga y Plataforma en Nanomedicina‐IBIMA Plataforma BIONAND Málaga 29590 Spain; ^3^ Basic Science Department Faculty of Health Universidad Industrial de Santander Bucaramanga 680001 Colombia; ^4^ Department of Pharmaceutical Science and Technology Faculty of Pharmacy Universitas Hasanuddin Makassar 90245 Indonesia

**Keywords:** crystal violet, dissolving, feedback system, microarray patches, silica

## Abstract

Dissolving microarray patches (DMAPs) represent an innovative approach to minimally invasive transdermal drug delivery, demonstrating efficacy in delivering both small and large therapeutic molecules. However, concerns raised in end‐user surveys have hindered their commercialization efforts. One prevalent issue highlighted in these surveys is the lack of clear indicators for successful patch insertion and removal time. To address this challenge, a color‐change‐based feedback system is devised, which confirms the insertion and dissolution of DMAPs, aiming to mitigate the aforementioned problems. The approach combines hydrophilic needles containing model drugs (fluorescein sodium and fluorescein isothiocyanate (FITC)‐dextran) with a hydrophobic poly(lactic acid) baseplate infused with moisture‐sensitive silica gel particles. The successful insertion and subsequent complete dissolution of the needle shaft are indicated by the progressive color change of crystal violet encapsulated in the silica. Notably, distinct color alterations on the baseplate, observed 30 min and 1 h after insertion for FITC‐dextran and fluorescein sodium DMAPs respectively, signal the full dissolution of the needles, confirming the complete cargo delivery and enabling timely patch removal. This innovative feedback system offers a practical solution for addressing end‐user concerns and may significantly contribute to the successful commercialization of DMAPs by providing a visualized drug delivery method.

## Graphical abstract

Uncover the potential  of dissolving microarray patches (DMAPs) in transdermal drug delivery. The paper introduces a color‐change feedback system, seamlessly melding hydrophilic needles with a hydrophobic baseplate to confirm DMAP insertion and dissolution. This innovation offers a visually guided drug delivery method, poised to transform the landscape and potentially ensure the triumphant commercialization of DMAPs.

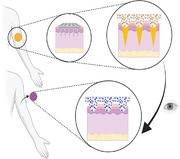

## 1 Introduction

Microarray patch (MAP) technology for transdermal drug delivery has expanded exponentially in recent times. During the early stages of development, MAP technology saw considerable success in the cosmetic industry, however, through both academic and industrial collaboration, a wide range of small and large therapeutic molecules have now been successfully delivered across the skin using MAPs.^[^ ^1^, ^4^ ^]^ Encouragingly, both patients and healthcare advisors have responded positively to MAP technology. For example, in a recent Phase I clinical trial, 98.6% of participants receiving an inactivated influenza vaccine through a dissolving MAP (DMAP) reported an overall positive experience.^[^ ^1^ ^]^ This is somewhat unsurprising given the pain‐free, minimally invasive nature of DMAP vaccine administration, in contrast to a conventional hypodermic needle and syringe. Furthermore, MAPs can be self‐applied, only requiring thumb pressure to achieve successful skin insertion.^[^ ^2^, ^3^ ^]^ Removing the need for administration by a healthcare professional has the potential to increase patient acceptance and improve vaccine coverage, particularly in low‐resource countries.^[^ ^4^ ^]^

Nevertheless, end‐user input remains vitally important to the commercial success of DMAP technology. Regulatory authorities have also offered valuable contributions to the field and have proposed requirements that should be considered before DMAPs can be accepted for clinical use. One such requirement is based on the ease and reliability of DMAP application to the skin. Indeed, several concept studies have developed feedback mechanisms as an indirect method of confirming successful skin insertion. This has included the use of a pressure‐indicating sensor film,^[^ ^5^ ^]^ a water‐filled reservoir,^[^ ^6^ ^]^ and snap‐based devices.^[^ ^7^ ^]^ Interestingly, to date, no all‐in‐one feedback mechanism has the ability to both confirm the successful insertion of the DMAP and indicate when the DMAP should be removed.

Therefore, in this study, a color‐change‐based system has been first designed and reported for the purpose of confirming DMAP insertion and dissolution. This has been achieved by incorporating a poly(lactic acid) (PLA) baseplate containing crystal violet dye encapsulated within silica into a DMAP. As a biopolymer, PLA is biodegradable and biocompatible, which is commonly used within the pharmaceutical industry. In addition, dye encapsulation using silica macro particles was selected as a viable method to retain crystal violet whilst preventing its skin absorption. The main principle behind this novel feedback mechanism, however, is based upon the color change of crystal violet when exposed to an aqueous media. This results in a color change from yellow to violet on the surface of the DMAP, producing an absorbance maximum of 590 nm and an extinction coefficient of 87 000 m ^−1^ cm^−1^. As successful insertion of the DMAP system into the skin results in exposure to interstitial fluid, hydration of the polymeric needles and the baseplate of the DMAP permit the color change of the dye. Not only would this provide a direct method of visibly confirming skin insertion, but the color change intensity could also relate to the dissolution of the drug‐loaded needles. This would indicate that the delivery cargo has been deposited successfully into the skin, addressing potential issues with drug dosing from regulatory authorities, healthcare providers, and the end‐user. The applicability of this feedback system on the delivery of both small and large therapeutic molecules such as nucleic acids and vaccines through DMAPs has been proved using two separate model compounds, fluorescein sodium (376 Da) and fluorescein isothiocyanate (FITC)‐dextran (150 kDa).^[^ ^8^ ^]^ Furthermore, cytotoxicity studies have been performed to illustrate the biocompatibility of this novel system.

This study has specifically discussed the prominent concerns highlighted by patients, healthcare professionals, and regulatory authorities in using DMAPs and, for the first time, provided innovative solutions, seeking to address the drawbacks associated with the current methods of drug administration. This certainly has imperative implications for the future commercialization of MAP technology.

## 2 Results and Discussion

### 2.1 Design and Characterization of a Moisture‐Indicating Baseplate

#### 2.1.1 Baseplate Formulation Design

**Figure 1:**
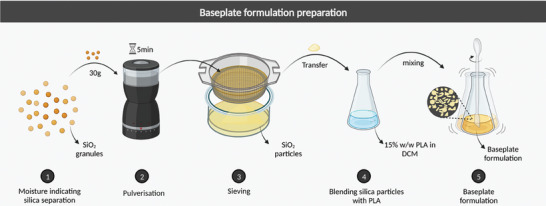
Schematic representation outlining the preparation of the baseplate formulations, B1 (15:1 PLA:M50) and B2 (20:1 PLA:M180).

Commercial moisture‐indicating silica pellets were first pulverized and granulated to particle sizes below 180 µm (M180) and 50 µm (M50). After removing the moisture that the silica absorbed from the air during the grinding process, two baseplate formulations, B1 and B2, were prepared by blending silica particles with 15% PLA (w/w) dissolved in dichloromethane (DCM) at a weight ratio of 15:1 (PLA: M50) and 20:1 (PLA: M180), respectively. The design flow of the baseplate formulation is shown in **Figure** **1**.

#### 2.1.2 Morphology of Baseplate Film

**Figure 2:**
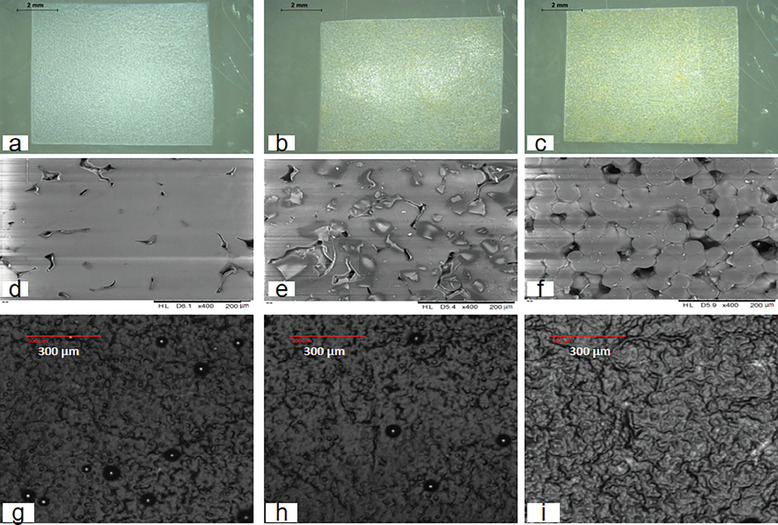
Morphology and tomography of the films made of a,d,g) PLA, b,e,h) PLA+M180, and c,f,i) PLA+M50 obtained by optical microscope, SEM, and acoustic microscope.

To facilitate characterization, a mold with a 1 cm^2^ insert made from silicone elastomer was used to cast films made using 300 µL baseplate material. This was the same volume that was used for the baseplate layer within the DMAPs. Using optical microscopy, scanning electron microscope (SEM), and acoustic microscopy imaging, the 1 cm^2^ films possessed a rough surface as shown in **Figure** **2**, with silica particles distributed uniformly amid PLA. The films were stored in the desiccator at room temperature prior to use to prevent moisture absorption.

#### 2.1.3 Mechanical Properties

**Figure 3:**
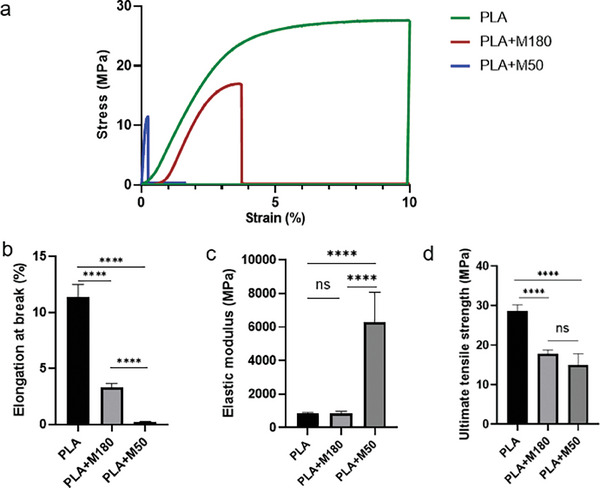
a) Representative stress/strain curves, b) elongation at break, c) elastic modulus, d) ultimate tensile strength, for films made of PLA, PLA+M180, and PLA+M50. **** represents (*p* < 0.0001) (means + SD, *n* = 3).

Tensile mechanical assessment was carried out with PLA, PLA mixed with M180 (20:1) and M50 (15:1) separately in a weight ratio of the baseplate formulations as described in Section 2.1.1. The addition of silica at sizes below both M180 and M50 reduced the adhesion of the PLA as the elongation at break diminished considerably (*p* < 0.0001) as shown in **Figure** **3**b. PLA+M50 (15:1) displayed higher stiffness with a much greater elastic modulus compared with PLA and PLA+M180 (20:1) (*p* < 0.0001) (Figure 3c). This indicated that the presence of a higher amount of silica particles decreased the elasticity of the material and is therefore less likely for the baseplate to withstand lengthwise tension.^[^ ^9^, ^10^ ^]^ Compared with pure PLA, the formulation with silica was more brittle as the ultimate tensile strength (UTS) was much lower (*p* < 0.0001). However, no significant difference was found for the UTS of both PLA+M180 and PLA+M50, which suggested that the maximum stress that the two formulations could withstand while being stretched was less affected by the load of silica or its particle size.

#### 2.1.4 Thermal Events

To evaluate the residue of DCM in the baseplate formulation, TGA and gravimetric analysis were performed. After being placed in an oven at 50 °C for 1 h, the weight loss on drying was less than 0.05%, confirming that the amount of residual solvent in the formulations was acceptable according to Q3C (R6) of ICH.^[^ ^10^, ^11^ ^]^ The thermograms and thermal events of the baseplate and its individual composition are detailed in Figure S1, Supporting Information and **Table** **1**, respectively. Pure silica and PLA+M50 (15:1) displayed an endothermic peak of desolvation due to the water evaporation from silica which was absorbed from the air during the weighing process. The melting temperatures of PLA, PLA dissolved in DCM after drying, PLA+M180 (20:1), and PLA+M50 (15:1) were in the range of reported values of around 170 °C. It was observed that PLA, after being dissolved in DCM and then solidified by solvent evaporation, showed a higher weight loss of 15.2%. The melting and decomposition endotherm of PLA both showed two peaks at 164.67 & 175.56 °C and 325.50 & 368.00 °C, respectively. This phenomenon might be attributed to the formation of multiple crystalline states or the impact of inorganic additives or contaminants.^[^ ^12^ ^]^ The absence of an endothermic peak for DCM and no weight loss presented near its boiling point from TGA and DSC indicated that the solvent was not present in the baseplate material.

**Table 1:** Thermal event and the corresponding DSC peaks and TGA weight loss for pure silica, pure PLA, PLA dissolved in DCM, and the two baseplate films, PLA:M50 (15:1) and PLA:M180 (20:1).

Thermal event	Pure silica		PLA		PLA (DCM)		PLA+M50		PLA+M180	
	Temp [°C]	WL [%]	Temp [°C]	WL [%]	Temp [°C]	WL [%]	Temp [°C]	WL [%]	Temp [°C]	WL [%]
Desolvation	98.52	11.89	‐	‐	‐	‐	93.77	4.77	‐	‐
Melting	‐	‐	164.67 175.56	2.46	174.89	15.20	176.00	8.91	176.40	3.85
Decomposition	‐	‐	325.56 368.00	50.4	363.56	52.20	365.56	73.3	367.28	74.6

#### 2.1.5 Chemical interactions

The infrared (IR) spectra of the baseplate materials and their compositions are shown in Figure S2, Supporting Information, with key regions highlighted in **Table** **2**. Using FTIR analysis, PLA dissolved in DCM did not show any DCM peaks, reflecting that DCM had fully evaporated from the polymer. Importantly, this was consistent with TGA. In the baseplate material, the absorption peak of the silicon‐oxygen bond was observed at 1072 cm^−1^, but no absorption peak of the crystal violet was found, possibly due to the low amount of dye (<0.3%) in the silica.^[^ ^13^, ^14^ ^]^ No chemical shifts in the formulation were found when compared with pure material spectra, indicating that there are no detectable chemical interactions occurring within the formulation.^[^ ^15^ ^]^

**Table 2:** Vibrational modes of interest for pure silica, pure PLA, PLA dissolved in DCM, the two baseplate films, PLA:M50 (15:1) and PLA:M180 (20:1), and crystal violet dye.

Vibrational modes	Wavenumber [cm^−1^]					
	Crystal violet	PLA	PLA (DCM)	PLA+M50	PLA+M180	Silica
ν(O─H)	‐	2668	‐	3673	‐	‐
ν(C─H)	2913	2956 2903	2967 2892	2967 2892	2961 2892	‐
ν(C═O)	‐	1751	1747	1751	1750	‐
ν(C─O)	‐	1075	1072	1066	1074	‐
ν( 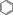 )	1576	‐	‐	‐	‐	‐
ν(C─N)	1157 1352	‐	‐	‐	‐	‐
ν(Si─O─Si)	‐	‐	‐	1075	1075	1072

#### 2.1.6 Wettability

Depending on the hydrophilicity of the molecule, the delivery efficiency of the active pharmaceutical ingredient can be affected by the physicochemical properties of the baseplate. This is particularly relevant with hydrophilic drugs as it is likely that drugs loaded into the needles themselves tended to migrate into the baseplate layer if hydrophilic material was used to fabricate the baseplate.^[^ ^16^, ^17^ ^]^ To prevent this, hydrophobic baseplates have been developed.^[^ ^16^, ^17^ ^]^ The wettability of the DMAP baseplate was studied by measuring the contact angle of water resting on films made of PLA+M180 (20:1), PLA+M50 (15:1), and polyvinylpyrrolidone (PVP). PVP is a commonly used hydrophilic baseplate within DMAP design and therefore was used as a control in this work. A static sessile drop method of the goniometer was applied to 3 different locations on each film, with the results shown in Figure S3, Supporting Information. The contact angle for PLA, PLA+M180 (20:1) and PLA+M50 (15:1) films was observed to be 58.42 ± 5.36°, 46.05 ± 9.97° and 54.14 ± 4.68° (*n* = 3), respectively. Compared with the PVP‐based film (contact angle = 42.73 ± 3.05°, *n* = 3), the PLA‐based films were less easily wetted by water. Interestingly, the addition of both M50 and M180 silica to PLA decreased the contact angle when compared to PLA alone. This suggests that silica might have a counteractive effect in hydrophobic interactions.

### 2.2 Fabrication and Characterization of FITC‐Dextran and Fluorescein Sodium DMAP with Moisture‐Indicating Baseplate

#### 2.2.1 Fabrication of DMAPs with Moisture‐Indicating Baseplate

**Figure 4:**
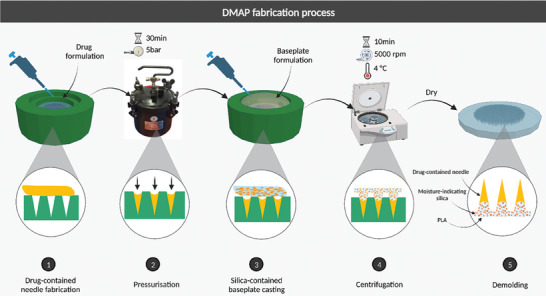
Schematic illustration of the fabrication process of DMAPs. The needle layer was formed by pressing the formulation into the needle cavities of the microneedle mold with a pressure of 5 bar in the pressure chamber for 30 min. After 24 h, the baseplate layer was cast onto the needle layer with 300 µL of baseplate material at a centrifuge speed of 5000 rpm for 10 min. The MAPs were demolded after 3 h of solvent evaporation in the fume hood.

The formulations of the needle layer and baseplate layer of FITC‐dextran and fluorescein sodium DMAPs are displayed in **Table** **3**. The fabrication of DMAPs followed a two‐step process, as depicted in **Figure** **4**. Molds consisting of 600 pyramidal needle holes 750 µm in depth, 300 µm in base width, and 50 µm in interspacing within an area of 0.76 cm^2^ were used in this study.

**Table 3:** Summary of formulations for FITC‐dextran 150 kDa and fluorescein sodium DMAPs.

Type of DMAP	Composition of needle layer [% w/w]					Composition of baseplate layer [% w/w]		
	(40% w/w) PVP	(40% w/w) PVA	Deionized water	FITC‐dextran	Fluorescein sodium	M50	M180	(15% w/w) PLA
FITC‐dextran 150 kDa	37.5	37.5	24	1	‐	6.25	‐	93.75
Fluorescein sodium	39.75	39.75	20	‐	0.5	‐	4.76	95.24

#### 2.2.2 Morphology of DMAPs

**Figure 5:**
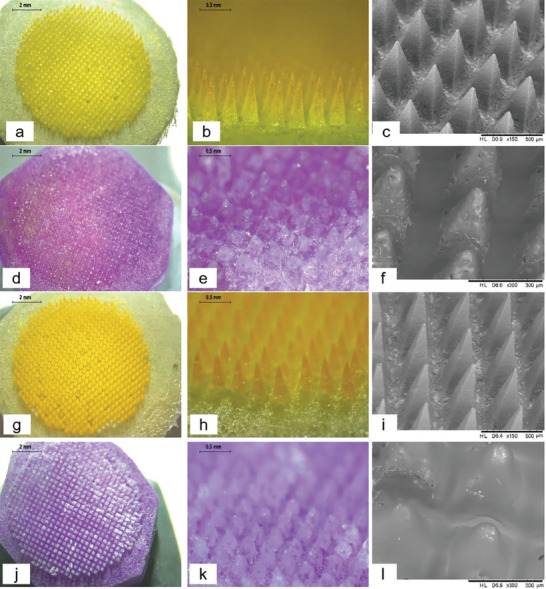
a–f) Morphology of DMAPs of FITC‐dextran and g–l) fluorescein sodium a–c,g–i) before and d–f,j–l) after needle dissolution in PBS obtained by optical microscopy and SEM.

**Figure 6:**
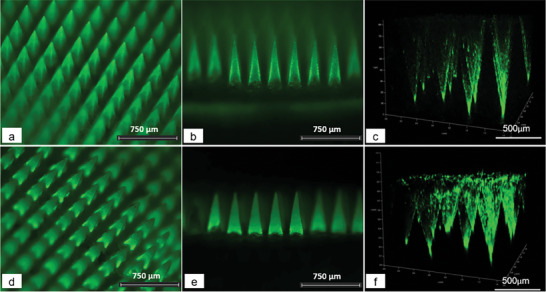
Representative fluorescence images and Z‐stack images of a–c) DMAP of FITC‐dextran 150 kDa and d–f) fluorescein sodium obtained by fluorescence microscope and multiphoton microscopy.

The morphology of DMAPs before and after dissolution was visualized using optical microscopy and scanning electron microscopy (SEM). When placed in phosphate‐buffered saline (PBS) (pH 7.4), the needle tips within both DMAPs disappeared in 3 h, with cone‐shaped pedestals remaining (Figure 5d–f,j–l). Furthermore, a distinct color change from buff to red‐purple (FITC‐dextran) and blue‐purple (fluorescein sodium) could be observed within the respective DMAP baseplates. Before dissolution, the needles of the patch presented a sharp tip and, after dissolution, the cone‐shaped pedestals on which the needle tips were mounted remained on the backing layer where silica gel particles were seen inlaid and dispersed as shown by SEM images. The distribution of FITC‐dextran and fluorescein sodium in the needles was examined using fluorescence microscopy. As shown in **Figure** **6**a–e, the fluorescence signals of the two DMAPs were concentrated at the needle tip and needle shafts only, indicating that the two compounds were not distributed in the backing layer. These results suggested that the baseplate made of PLA could prevent the migration of drugs from the needle to the baseplate to some extent. The images captured from multi‐photon scanning microscope also demonstrated high signal intensity at the needle tip and shaft, with no fluorescence generated from the baseplate (Figure 6c,f).

#### 2.2.3 Mechanical Strength of DMAPs

**Figure 7:**
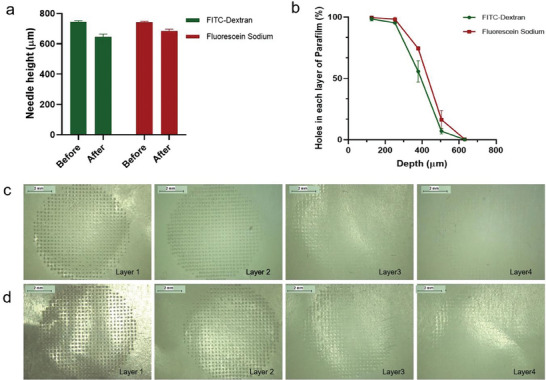
Results of the a) height reduction test (means + SD, *n* = 5) and b) Parafilm M insertion test (means ± SD, *n* = 5) of FITC‐dextran 150 kDa and fluorescein sodium DMAPs. Images of holes created by c) FITC‐dextran 150 kDa DMAP and d) fluorescein sodium DMAP in each layer of Parafilm M.

DMAPs individually loaded with either FITC‐dextran 150 kDa or fluorescein sodium exhibited a height reduction percentage of less than 20%, which suggested that the needles had desirable hardness and mechanical strength to penetrate the skin^[^ ^18^ ^]^ (**Figure** **7**a). Eight layers of Parafilm were folded to reach a total height of ≈1000 µm, with the thickness for each sheet about 126 µm. Upon insertion, the holes created by the DMAP in each layer were counted under the microscope and the hole percentage was calculated, as shown in Figure 7c,d. Layers with a penetration rate of more than 20% were deemed to be successfully penetrated.^[^ ^19^ ^]^ The insertion depth was assessed by plotting the thickness of the Parafilm layer as X axial and the percentage of total needles penetrating each layer as Y axial (Figure 7b). The insertion depth of the two DMAPs was between ≈378 and 504 µm, thus exceeding the thickness of the stratum corneum (SC) and successfully reaching the viable epidermis for systemic drug delivery.^[^ ^20^, ^21^ ^]^

#### 2.2.4 Skin Insertion Ability of DMAPs

**Figure 8:**
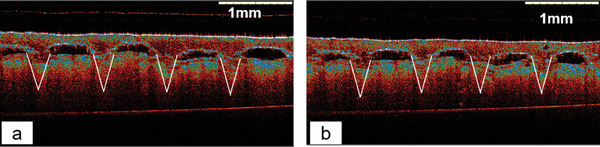
Representative OCT images of insertion test in the skin for a) FITC‐dextran 150 kDa DMAP and b) fluorescein sodium DMAP.

The penetration of DMAPs through full‐thickness neonatal porcine skin was also used to evaluate insertion ability. The results of this test are shown in **Figure** **8**. The total insertion depth of DMAP loaded with FITC‐dextran 150 kDa and fluorescein sodium were 536.7 ± 19.6 µm (*n* = 6) and 601.5 ± 20.2 µm (*n* = 6), respectively. The total length of the needle was around 750 µm, so the insertion percentage of the needles was calculated to be 71.47% for FITC‐dextran 150 kDa DMAP and 80.2% for fluorescein sodium DMAP, which aligned with work performed previously.^[^ ^22^ ^]^

### 2.3 Ex Vivo DMAP Dissolution and Baseplate Color Change

**Figure 9:**
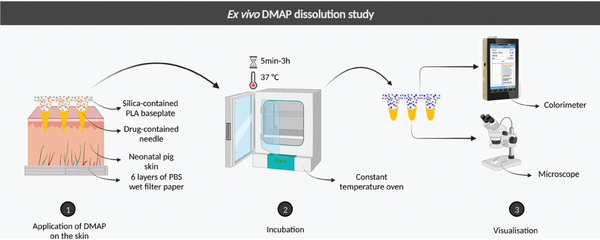
Schematic illustration of the ex vivo dissolution study using FITC‐dextran 150 kDa and fluorescein sodium DMAPs.

Dissolution testing of FITC‐dextran 150 kDa and fluorescein sodium DMAPs was conducted to determine the time in which the DMAPs should be removed from the skin. The DMAPs were applied to excised neonatal porcine skin using a force of 32 N applied by texture analyses, with the skin placed on top of the tissue paper wet with PBS (pH 7.4) and maintained at a temperature of 37 °C. The needle and surface of the DMAP were visualized at pre‐determined time intervals. The length of the needle was recorded under the microscope while the color of the baseplate was described in green‐red, blue‐yellow, and light scale with a handheld colorimeter. The process is demonstrated in **Figure** **9**.

**Figure 10:**
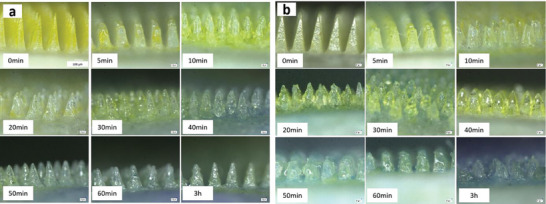
Height of a) FITC‐dextran 150 kDa and b) fluorescein sodium DMAP needles obtained by optical microscopy after insertion into neonatal porcine skin.

The height of the needles was measured under the microscope 5 min after skin insertion and every 10 min thereafter until the length no longer changed. The dissolution process of the two DMAPs is shown in **Figure** **10**. As depicted in Figure 10a, the needle tips of FITC‐dextran DMAP fully dissolved within the first 5 min after insertion, after which the needle shaft underwent a deformation until it was fully dissolved. The height of the needle did not change after ≈30 min, with a conical‐shaped needle base remaining on the insoluble PLA baseplate. For fluorescein sodium DMAP, the tip of the needle had also fully dissolved after 5 min. The needle shaft also contained fluorescein sodium dissolved in ≈50 min, with the needle height remaining unchanged after ≈60 min (Figure 10b). The different dissolution times for the two DMAPs might be a result of varied amounts of PVA, PVP, and model drug in the formulation. For fluorescein sodium, a larger quantity of PVA and PVP was used to manufacture the DMAPs compared with FITC‐dextran 150 kDa considering the physical properties and simulation purpose of the molecular in this study. Consequently, it took longer for the fluorescein sodium DMAPs to fully dissolve. Importantly, the dissolution span obtained in this study is in good agreement with the results from the DMAPs made out of PVA and PVP in previous work.^[^ ^23^, ^24^, ^25^, ^26^ ^]^

The color of the baseplate surface of the DMAPs was recorded using a handheld colorimeter, which expressed color in three dimensions (L*, a*, and b*). The a* axis was relative to the green‐red opponent colors, with negative numbers toward green and positive toward red. The b* axis represented the blue‐yellow opponents, with negative values toward blue and positive toward yellow. The perceptual lightness was described in L* value, defining black at 0 and white at 100.^[^ ^27^, ^28^ ^]^ The color difference (Δ*E*) between 0 min and each time point was automatically obtained from the spectrophotometer as the color of the baseplate before DMAP insertion into the skin was set to be the reference.

**Figure 11:**
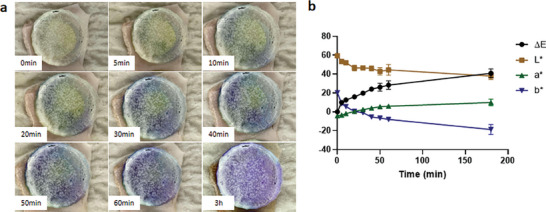
a) Images of the baseplate of FITC‐dextran DMAP and b) the result of color measurement during ex vivo dissolution study (means ± SD, *n* = 4).

As shown in **Figure** **11**, 5 min after insertion of the FITC‐dextran 150 kDa DMAP, Δ*E* was measured to be 9.92 ± 0.37 (*n* = 4). With a Δ*E* value greater than 2.3, this color change is noticeable to the human eye. Furthermore, the change in Δ*E* indicates the successful insertion of the DMAP into the skin, given that the color change is a result of the deprotonation of the crystal violet by interstitial fluid migrating from the needle to the baseplate.^[^ ^29^ ^]^ Further hydration of the DMAP over time resulted in the color of the backing layer shifting from yellow‐green to blue‐purple, yielding an increase in Δ*E* and a* and a decrease in L* and b* (Figure 11b). After 30 min, a discernible purple color was displayed on the baseplate of the DMAP, corresponding to a value of 19.78 ± 2.06 (*n* = 4) in Δ*E*. This correlated with the optical microscope images in Figure 10, in which dissolution of the needles in the DMAP occurred within 30 min. Therefore, integrating the results of the needle height and the color change in this test, the patch could be removed from the skin when a significant purple color was present on the baseplate, which in this case occurred after 30 min.

**Figure 12:**
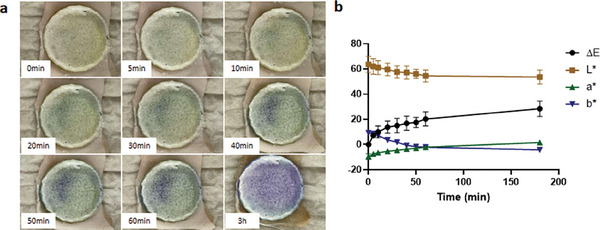
a) Images of the baseplate of fluorescein sodium DMAP and b) the result of color measurement during ex vivo dissolution study (means ± SD, *n* = 4).

The surface of the baseplate of fluorescein sodium DMAP was photographed and measured at each time point (**Figure** **12**). A value of 7.22 ± 0.94 (*n* = 4) in Δ*E* was given by the colorimeter 5 min after the application of the DMAP to the excised neonatal porcine skin, indicating the successful insertion of the fluorescein sodium DMAP into the skin. A blue‐purple color change appeared gradually on the surface of the baseplate, where a prominent violet was observed at 60 min, with a Δ*E* of 20.25 ± 5.58 (*n* = 4). As the color altered from yellow‐green to blue‐purple, the value of a* steadily increased, the value of b* progressively decreased, and the value of L* slightly decreased, as displayed in Figure 12b. The complete dissolution of the needles was investigated to be 60 min, which corresponded to the distinct color change of the backing layer. This implied the timing of removing the DMAP.

Upon insertion, the needle layer blocked direct contact between the baseplate and the skin. As the water‐soluble shaft of the needle dissolved, interstitial fluid from the skin had a greater chance of migrating to the backing layer and being absorbed by the silica, triggering the color change of the dye within the silica. The distinctive color change of the baseplate for FITC‐dextran 150 kDa DMAPs occurred in 30 min, whilst the same color change occurred after 1 h with fluorescein sodium. In addition, the Δ*E* values obtained in this study were significantly greater for FITC‐dextran 150 kDa when compared to fluorescein sodium DMAPs (*p* < 0.05). This outcome could be due to the finer size and higher proportion of silica particles in the baseplate formulation of FITC‐dextran 150 kDa DMAPs leading to the faster fluid absorption of silica and a more intensive deprotonation.^[^ ^30^ ^]^ This was in line with the intention of our formulation design. In order to simulate the short duration of the color change, a fast dissolution DMAP was developed with FITC‐dextran 150 kDa loaded in the needle layer in represent of the transdermal administration of macromolecules such as vaccines and mAbs, where a short vaccination time to facilitate both patients and healthcare professionals was preferred. By comparison, a moderate color change and a longer release profile of DMAP were obtained using fluorescein sodium as an example for small molecules. For both DMAPs, the dissolution of the needle was associated with the color transformation of the baseplate surface, signifying that the progression of DMAP dissolution inside the skin could be revealed by a simple visual user‐feedback mechanism. This technology would be straightforward enough to be comprehended through a patient information leaflet along with the product in the commercialization scenario.

### 2.4 In Vitro Delivery of FITC‐Dextran 150 kDa and Fluorescein Sodium

**Figure 13:**
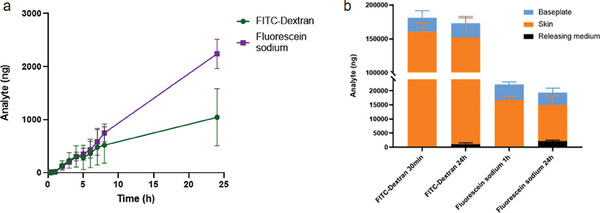
a) In vitro delivery of FITC‐dextran 150 kDa and fluorescein sodium DMAP through the skin (means ± SD, *n* = 3) and b) the amount of analyte being delivered to the releasing medium, deposited in the skin and remaining on the baseplate after 30 min (FITC‐dextran 150 kDa), 1 h (fluorescein sodium) and 24 h (means + SD, *n* = 3).

The in vitro delivery efficiency of FITC‐dextran 150 kDa and fluorescein sodium DMAPs through neonatal porcine skin was evaluated using a Franz diffusion system. The model drugs released into the receptor compartment at each time point were quantified using a plate reader. FITC‐dextran 150 kDa and fluorescein sodium that were both deposited in the skin and remained on the baseplate at full dissolution time (30 min for FITC‐dextran 150 kDa DMAP and 1 h for fluorescein sodium DMAP) and 24 h were extracted and determined. As shown in **Figure** **13**a, a steady increase in concentration within the receiver medium was observed for both DMAPs in 24 h. The release of both compounds from the DMAPs after 24 h still showed an upward trend instead of reaching a plateau, which may be a result of continuous delivery of drugs from the depot of polymer and drug matrix retained intradermally.^[^ ^31^, ^32^, ^33^, ^34^ ^]^ For FITC‐dextran 150 kDa DMAPs, the needles were completely dissolved at 30 min, when most of the FITC‐dextran 150 kDa resided in the skin (160.49 ± 12.60 µg) and minimal levels were detected in (0.0047 ± 0.0042 µg) the receiver medium by comparison. At 24 h, 1.04 ± 0.54 µg of FITC‐dextran 150 kDa was delivered into the receptor, with a slight decrease in FITC‐dextran 150 kDa present in the skin (151.06 ± 28.57 µg). There was no significant difference (*p* > 0.05) in the drug content within the backing layer at the 30 min (20.93 ± 10.20 µg) and 24 h (20.83 ± 9.96 µg) time points, which proves that keeping the DMAP in place for longer than 30 min would have no added benefit (Figure 13b). This is important for the end user and could ultimately reduce the patch wear time, potentially a key contributor to improving patient acceptability. Regarding fluorescein sodium DMAPs, the amount of fluorescein sodium released into the receptor at 1 h was 0.023 ± 0.025 µg, but reached 2.24 ± 0.27 µg over 24 h, with intradermal fluorescein sodium deposition decreasing from 16.64 ± 2.14 µg at 1 h to 12.77 ± 3.19 µg at 24 h. The residual fluorescein sodium on the baseplate decreased from 5.52 ± 0.94 µg at 1 h to 4.33 ± 1.57 µg at 24 h (*p* > 0.05). These results indicate that DMAP removal at 1 h is still feasible in view of the comparable drug amount that remained on the baseplate between the 1 and 24 h time points.^[^ ^35^, ^36^ ^]^

### 2.5 Biocompatible Study

#### 2.5.1 Dye Retention Ability within Silica

Despite the documented medical use of crystal violet, it was still deemed essential to ensure that the silica had the ability to retain the dye and no crystal violet was delivered to the skin together with the drug.^[^ ^37^ ^]^ Therefore, the dye retention test was carried out with the silica particle. The silica was pore‐sized with a vast network of interconnecting orifices in the diameters of 20‐30 Å.^[^ ^14^ ^]^ These pores are known to have a strong affinity for moisture and dye owing to physical adsorption and capillary condensation.^[^ ^38^, ^39^ ^]^ The dye retention ability of the silica has been evaluated by quantifying the amount of crystal violet in the suspension of silica particles and in the skin after the dissolution of DMAPs.^[^ ^40^, ^41^ ^]^

A calibration curve for crystal violet from the solution shown in Figure S4a, Supporting Information with a liner range from 0.125 to 4 µg mL^−1^ was obtained. After being suspended in PBS (pH 7.4), the silica was centrifuged. As shown in Figure S4b, Supporting Information, the solution was crystal clean in the supernatant with bluish silica only at the bottom of the tube resulting from the hydration of the dye trapped inside. From the scan of the supernatant by UV spectrometer, no absorption was observed and no dye above the detection limit of the analytical method was detected (Figure S4c, Supporting Information). The results indicated that the silica has a strong affinity to the dye, thereby avoiding the delivery of dye in the baseplate into the skin.

**Figure 14:**
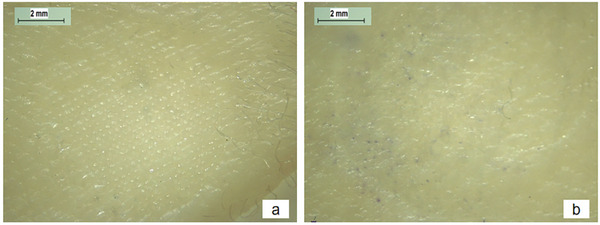
Images of skin after insertion of blank DMAPs loaded with a) dye encapsulated in silica and b) pure dye in the baseplates after 3 h.

The amount of crystal violet in the skin was extracted and quantified after insertion of blank DMAPs equipped with a PLA baseplate which was dispersed with dye encapsulated in silica or pure dye. The skin was visualized under the microscope after 3 h, as shown in **Figure** **14**. The skin inserted by DMAP loaded with dye encapsulated in silica in the backing layer did not result in perceptible dye on the surface, as shown in Figure 14a and the skin extraction did not yield any measurable absorbance at 592 nm from the UV spectrophotometer. While for the DMAP with a baseplate composed of pure dye suspended in PLA, there were discernible blue dots on the surface of the skin after the removal of the DMAP (Figure 14b). The concentration of crystal violet in the supernatant of the skin homogenate was calculated to be 0.13 ± 0.10 µg. This result indicated the essentiality of encapsulating the dye inside the silica.

#### 2.5.2 SiO_2_ Residue after Insertion

**Figure 15:**
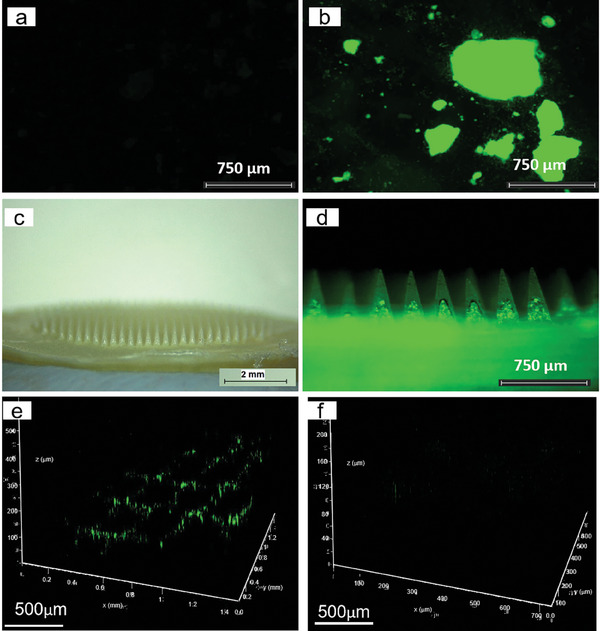
Images of silica a) before, b) after FITC labeling, and c,d) after being loaded to the blank DMAP. e) Z‐stack images obtained by multiphoton microscopy of blank DMAP with labeled silica in the baseplate being inserted into the skin and f) skin after the DMAP was removed after 3 h.

The silica particles were fluorescently labeled with FITC to track the location of the silica after insertion and evaluate potential silica migration to the skin upon dissolution of the DMAPs. Successful fluorescent labeling was confirmed by fluorescence microscopy (**Figure** **15**), as a strong fluorescent signal was obtained in the labeled silica powder under the appropriate excitation wavelength (Figure 15b). On the contrary, the non‐labeled silica powder did not produce any significant fluorescence under the same imaging conditions (Figure 15a). After confirming the successful labeling of the silica, DMAP with labeled silica in the baseplate was fabricated (Figure 15c). From the fluorescence microscopy images of the DMAPs containing labeled silica, the silica was observed to be evenly distributed in the baseplate, and no fluorescent signal was observed at the needle tip (Figure 15d).

Employing multiphoton microscopy, the presence of the labeled silica powder in the baseplate was further observed while the DMAP was inserted into full‐thickness neonatal porcine skin (Figure 15e). After the removal of the DMAP from the porcine skin at 3 h, there was no fluorescence signal in the skin, which suggested that no silica residue was left inside the skin after the insertion of the DMAP (Figure 15f). To provide a quantitative measurement of this, the skin homogenate after insertion with the DMAP was analyzed using a plate reader with an excitation wavelength of 485 nm and an emission wavelength of 520 nm. In the same experiment, skin treated with unlabeled silica baseplate DMAP was used as a control. The fluorescence signal of the skin homogenate showed fluorescence intensities below the detection limit (0.97 µg mL^−1^), which further pointed out a negligible transfer of the silica included in the baseplate to the skin as a humidity and insertion indicator.

#### 2.5.3 Cytotoxicity

**Figure 16:**
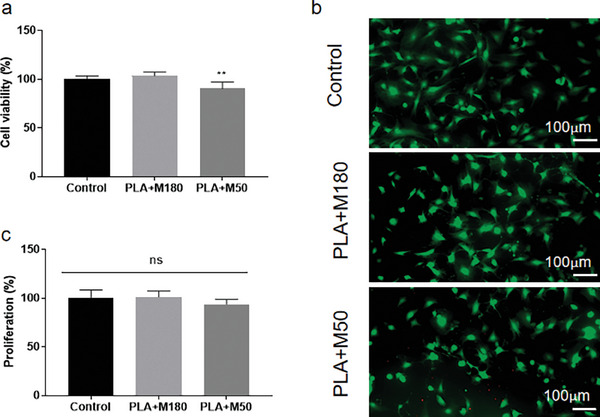
a) The percentage of viable cells after 72 h of culture with PLA+M180 and PLA+M50 DMAPs in MTT assay (means + SD, *n* = 6). b) Alive/dead staining of fibroblastic cells on control, PLA+M180, and PLA+M50 treated samples (green represents alive and red represents dead). c) Total DNA content of cells on control, PLA+M180 and PLA+M50 cultured for 72 h in PicoGreen assay (means + SD, *n* = 6). ** represents *p* < 0.001.

In this study, an MTT assay was used to determine any cytotoxic effects of the baseplate formulations on human dermal fibroblasts. As shown in **Figure** **16**a, the MTT test results showed that the percentage of cell viability of DMAPs with PLA+M180 (20:1) and PLA+M50 (15:1) baseplate were 103.50% and 90.36%, respectively, after 72 h. According to statistical analysis, the values between the control and sample treated with PLA+M50 (15:1) were significantly different, indicating the influence of the smaller particle and the higher amount of silica causing lower cell viability in the experiment (*p <* 0.001). Despite this significant difference, based on the classification of cytotoxicity level and the ISO 10993‐5, as described before,^[^ ^42^, ^43^, ^44^ ^]^ the sample containing PLA+M50 (15:1) was categorized as having no cytotoxicity (grade 1). On the other hand, the absence of red fluorescence following calcein/ethidium homodimer‐1 staining for cells treated with PLA+M180 (20:1) and PLA+M50 (15:1) DMAPs indicated the low quantity of extracellular nucleic acid released from dead cells with damaged plasma membranes (Figure 16b). The observation from Figure 16b, along with the results from Figure 16a, supported the fact that exposure of the cells to the silica and the polymer PLA did not compromise the viability or plasma membrane integrity of the fibroblast cells. From a clinical standpoint, the data suggested that PLA+M180 (20:1) and PLA+M50 (15:1) are unlikely to result in meaningful toxicity to the skin. In addition, PicoGreen assay was also conducted to further elucidate the impact of the formulation on the proliferation of healthy cells upon exposure to the formulation and the excipients for a 72‐h period. It was observed that exposure to PLA+M180 (20:1) and PLA+M50 (15:1) did not impact the overall proliferation of the cells (Figure 16c).

In this in vitro cytotoxicity study, three different tests were conducted to demonstrate the biocompatibility of silica with crystal violet suspended in DCM. The overall results confirmed that the baseplate formulations tested were considered as lacking cytotoxic properties to the fibroblast cells in the human skin. Additionally, the silica was unlikely to be delivered into the skin, as confirmed in Section 2.5.2. Therefore, the materials involved in fabricating this device could be deemed safe for human application.

## 3 Conclusion

In this study, a novel delivery efficiency feedback system for biphasic DMAPs based on a PLA baseplate containing crystal violet dye encapsulated within silica was successfully developed. The insertion of DMAPs into the skin was indicated by a color change discernible to the human eye in 5 min. Additionally, complete dissolution of the needles from DMAPs and subsequent drug release could be aligned with the obvious color change of the backing layer, thereby prompting the correct time to remove the patches. This has the potential to increase user acceptance and give them the confidence that the loaded dose has been administered. With the successful delivery of the model drugs fluorescein sodium and FITC‐dextran 150 kDa, the delivery efficiency feedback system demonstrates their potential employment in the delivery of both small molecules and biomacromolecules. Furthermore, cytotoxicity studies on human dermal fibroblasts illustrated the biocompatibility of the material used in this system, providing a foundation for its practical application in healthcare regulation. The novel system designed in our study may address some of the current questions highlighted by the end‐user and regulatory authorities and thus contribute to the future commercialization of DMAP technology.

## 4 Experimental Section

### Materials

PLA Ingeo Biopolymer 3D850 was purchased from NatureWorks (Minnesota, United States). Orange indicator silica pellets were obtained from Interra Global Corporation (Park Ridge, US). FITC, (3‐aminopropyl) triethoxysilane (APTES), DCM, PBS tablets (pH 7.3–7.5), poly(vinyl alcohol) (PVA) (9–10 kDa) and FITC‐dextran (150 kDa) were purchased from Sigma Aldrich (St. Louis, MO, USA). Fluorescein sodium was purchased from Fluka (Buchs, Switzerland). PVP Plasdone K‐29/32 (58 kDa) was obtained from Ashland (Kidderminster, UK). Ultrapure water was obtained from a water purification system (Elga PURELAB DV 25, Veolia Water Systems, Dublin, Ireland).

### Design of Moisture Indicating Baseplate Formulation

Orange moisture indicator silica pellets (30 g) were crushed with a pulverizer (Kitchen Perfected Grinder, Singh bargain Ltd., London, UK) for 5 min, then the crushed silica was sieved through a 180 µm stainless steel woven wire mesh (Endecotts, London, UK). The particles with a size less than 180 µm after screening were collected and named as M180. M180 was pulverized for another 3 min and sieved through a 50 µm‐sized screen. Sieved products with a size of fewer than 50 µm were collected and named M50. Both M180 and M50 were dehydrated at 80 °C for 30 min and stored in the desiccator before use. A stock solution made out of 15% w/w of PLA was prepared by dissolving 6 g of PLA in 34 g of DCM overnight, stirring twice with a spatula. Moisture indicating baseplate material 1 (B1) was formulated by mixing 15% w/w PLA and M50 uniformly at a weight ratio of 15:1. Moisture indicating baseplate material 2 (B2) was prepared by mixing 15% w/w PLA and M180 evenly at a weight ratio of 20:1.

### Fabrication of Baseplate Film for Characterization

Baseplate films were prepared by using the same formulation as outlined in “Design of Moisture Indicating Baseplate Formulation”. 300 µL of each formulation was added to the mold and was centrifuged at 3500 rpm for 15 min and maintained at 4 °C. The molds containing the cast material were placed in a fume hood to dry for 3 h after which the films were demolded. The surface morphology and tomography of the films were observed using the Leica EZ4W stereo optical microscope (Leica Microsystem Ltd., Milton Keynes, UK), SEM (Hitachi TM3030, Hitachi High Technologies America Inc., USA), and scanning acoustic microscopy (SAM, Nikon Eclipse LV150N, Leuven, Belgium) equipped with an easySAM objective (Kibero GmbH, Saarbrücken, Germany).

### Mechanical Properties Evaluation

A glass Petri dish with a diameter of 6 cm was used to prepare films for baseplate mechanical assessment. Briefly, 5 mL of each formulation was injected into the Petri dish and was turned gently until the blends coated the bottom. Upon drying, the film was cut into strips in the geometry of rectangular (5 cm × 1 cm). Tensile mechanical testing of the films was carried out on the TA.XTplus texture analyzer (Stable Micro Systems, Surrey, UK) with a strain height of 3 cm at a constant extension speed of 10.2 mm min^−1^. The width and the length of the film were measured using a caliper to calculate the stress area and were inputted into the Exponent Stable Micro System. Both ends of the film were fixed in the middle of the clamp. Force displacement curves were recorded and UTS and elongation at failure was obtained from the curve. Moreover, the elastic modulus was calculated as the slope of the initial linear part of the stress/strain curve.^[^ ^10^, ^45^ ^]^

### Thermal Analysis

The residue of solvent in the baseplate was evaluated by weight analysis using thermogravimetric analyzer (TGA) (TGA Instruments, New Castle, USA), oven (Genlab Ltd., England, UK), and differential scanning calorimetry (DSC, TA Instruments, New Castle, USA).

In gravimetric analysis, the film was weighed before and after being placed in an oven for 1 h at 50 °C. A constant weight was considered to be achieved if the weight change was less than 0.25%.^[^ ^46^ ^]^ For TGA analysis, 5–10 mg of each sample was carefully weighed and placed inside an open aluminum pan and placed on the automatic loader of the TGA system. The sample was heated in ramp mode with a temperature gradient of 10 °C min^−1^ under nitrogen flow from 25–400 °C, and the weight variation was recorded and analyzed using TA Instruments Universal Analysis 2000 software. For DSC analysis, 5–10 mg of each sample was weighed and placed inside an aluminum pan and sealed with an aluminum lid. The sample was then placed on top of the heating stage side by side with the reference crucible. The DSC was run in ramp mode with a heating rate of 10 °C min^−1^ under nitrogen flow from 25 to 400 °C.

### FTIR Analysis

The chemical structure of the baseplate material was analyzed by FITR. PerkinElmer Spectrum Two FT‐IR Spectrometer (PerkinElmer, Buckinghamshire, UK) with Pike MIRacle ATR attachment (Pike Technologies, Wisconsin, USA) was used to measure the absorbance of the materials in the infrared region. The individual sample was fitted into the ATR diamond head and secured using the instrument's screw mechanism. The absorbance between the wavenumber of 750 to 4000 cm^−1^ was measured. The acquired spectra were processed and analyzed using Spectrum IR software. The results were plotted as transmittance versus wavenumber graph after normalization.

### Wettability

The wettability of the baseplate was evaluated by measuring the contact angle of water on hydrophobic baseplate films made of PLA‐based material and hydrophilic baseplate films composed of PVP‐based material. PVP‐based baseplate material was formed by blending 30% w/w of PVP (360 kDa) with 1.5% (w/w) of glycerin and 68.5% (w/w) of deionized water. The PVP‐based baseplate was commonly used and worked as a control in this study.^[^ ^19^, ^23^, ^25^, ^47^ ^]^ The contact angle was measured by the static sessile drop method using an Attension Theta Lite optical goniometer (Biolin Scientific, Manchester, UK) with an image resolution of 752 × 582 pixels, 50 fps video recorder, and ±0.1° accuracy. The drop volume was set to 4.0 µL with a drop rate of 1 µL s^−1^ and a dispense rate of 20 µL s^−1^. Once deposited, the drop was allowed to stabilize for 30 s to reach its final static state on the surface. Then the drop profile was monitored and analyzed by the software embedded. Measurements were done in triplicate.

### Fabrication of DMAPs with Moisture Indicating Baseplate

The stock solution for polymers (PP2) was prepared by mixing equal quantities of 40% w/w PVP and 40% w/w PVA. The formulation for FITC‐dextran 150 kDa (F1) was composed of 75% w/w PP2, 1% w/w FITC‐dextran 150 kDa, and 24% w/w deionized water. The formulation for fluorescein sodium (F2) consisted of 79.5% w/w PP2, 0.5% w/w fluorescein sodium and 20% w/w deionized water. The formulation was accurately weighed and was mixed in the Speedmixer DAC 150.1 FVZ‐K (GermanEngineering, Hauschild & Co. KG, Hamm, Germany) at 3500 rpm for 3 min. To prepare the first layer, an excess blend was transferred on top of the mold and evenly spread with a spatula. The formulation was then forced to fill into the needle cavities under a pressure of 5 bar in the pressure chamber for 30 min. The excess was removed by scraping the surface of the mold using a spatula. The first layer was dried for 24 h at ambient temperature to evaporate water from the formulation. The baseplate material B1 (300 µL) was added on the top of the first layer of F1 and centrifuged at 5000 rpm for 10 min at 4 °C to prepare FITC‐dextran DMAP. The same procedure was applied to prepare fluorescein sodium DMAP with B2 and F2. The baseplates for the two DMAPs were left to solidify in a fume hood at room temperature for 3 h for solvent evaporation and then demolded manually afterward.

### Morphology Observation

The morphology of DMAP was evaluated by Leica EZ4W stereo optical microscope, EVOS FL fluorescence microscope (Thermo Fisher Scientific, Waltham, USA) with a channel of transparent and FITC, SEM, and Leica TCS SP8 multi‐photon scanning microscope (Leica Microsystems Ltd., Milton Keynes, UK).

Stereo fluorescence images for DMAPs were collected using a multi‐photon scanning microscope equipped with an upright DM6 microscope body and a motorized stage. Leica Application Suite X software (3.5.7.23225) was used for image acquisitions. FITC‐dextran 150 kDa and fluorescein sodium loaded in the DMAPs were excited with 900 nm laser lines from the Mai Tai Deep See Mode‐Locked laser system (Newport‐Spectra Physics, UK). Fluorescence emission was collected via HyD GaAsP‐spectral internal detector for FITC‐dextran 150 kDa and fluorescein sodium between 496 and 549 nm. Optical sections of the specimen between 300 and 1000 µm deep into the tissue were collected with the distance between the images in the Z‐stack between 5 and 10 µm as appropriate. Image analysis was performed in the Leica Application Suite X software (3.7.020979).

### Mechanical Strength Determination

To ensure the successful insertion of the DMAP intradermally, mechanical strength and their insertion capability were investigated using a TA.XT2 Texture Analyser (Stable Micro Systems., Ltd., Haslemere, UK) as previously reported.^[^ ^26^ ^]^ To investigate mechanical strength, the DMAP was first put on a flat aluminum stage with needles facing downward. A force of 32 N was applied on the baseplate of the DMAP for 30 s at a speed of 0.5 mm^−1^ s. The needle height before (*H*_0_) and after (*H*_d_) compression were measured and recorded, and the height reduction was calculated according to Equation (1).

(1) $$\mathrm{Height}\;\mathrm{reduction}\;\left(\%\right)=\frac{H_{0}\;-\;H_{d}}{H_{0}}\times 100\%$$

The same compression procedure was employed to conduct the insertion, with the solid surface replaced with eight layers of Parafilm M. Upon insertion, the needles were taken out and the holes inside each layer of the artificial membrane were counted under the microscope. The percentage of holes in each layer relative to the number of needles on the array was determined using Equation (2). According to the model, the height of each Parafilm M layer was 126 µm, and layers with a penetration rate of more than 20% were considered fully penetrated.^[^ ^48^, ^49^ ^]^

(2) $$\mathrm{Holes}\;\mathrm{in}\;\mathrm{parafilm}\;\left(\%\right)=\frac{\mathrm{Number}\;\mathrm{of}\;\mathrm{holes}\;\mathrm{observed}}{\mathrm{Number}\;\mathrm{of}\;\mathrm{MN}\;\mathrm{in}\;\mathrm{total}}\times 100\%$$

### Skin Insertion Ability Evaluation

The insertion capability of DMAP in ex vivo full‐thickness neonatal porcine skin was performed using EX‐101 optical coherence tomography (OCT) microscope (Michelson Diagnostics Ltd., Kent, UK). The skin was prepared as described in previous work.^[^ ^22^ ^]^ Using thumb force, the DMAP was inserted into the skin and was visualized under OCT microscope. The insertion percentage was calculated by dividing the insertion depth by the total needle length.

### Ex Vivo DMAPs Dissolution Study

Both dissolving DMAPs loaded with FITC‐dextran 150 kDa and fluorescein sodium were inserted into full‐thickness neonatal porcine skin and placed on a moistened six‐layer tissue paper to keep the skin hydrated and simulate the subcutaneous environment. The unit was then placed in an incubator at a fixed temperature of 37 °C. The DMAPs were first removed from the skin after 5 min and the length of the needles was measured, followed by every 10 min until the needles had dissolved. At each time point, the height of the needles was recorded, and the color of the baseplate was determined by a spectrophotometer kindly supported by X‐Rite (Grand Rapids, Michigan, United States). CIELAB color space, which expressed color in three dimensions (L* for perceptual lightness, a* and b*) for the four unique colors of human vision (red, green, blue, and yellow), was used in this study to describe the color of the baseplate. The a* axis was relative to the green‐red opponent colors, with negative numbers toward green and positive toward red. The b* axis represented the blue‐yellow opponents, with negative values toward blue and positive toward yellow. The a* and b* values were unbounded and were clamped in the range of −128 to 127 for practical reasons. The lightness value L* defined black at 0 and white at 100. CIELAB was intended as a perceptually uniform space, where a given numerical change in three values corresponds to a similar perceived change in color. The color change (difference) was defined as distance metric Δ*E*, as calculated by Equation (3), where a difference of 2.3 stood for a “just noticeable difference” (JND) which was noticeable and detectable to the perception of human eyes. The larger the Δ*E* number, the more significant the difference between the two colors.^[^ ^50^ ^]^

(3) $$\Delta E_{t}^{*}=\sqrt{{\left(L_{t}^{*}-L_{0}^{*}\right)}^{2}+{\left(a_{t}^{*}-a_{0}^{*}\right)}^{2}+{\left(b_{t}^{*}-\;b_{0}^{*}\right)}^{2}}$$

The baseplate was measured before insertion and the result was recorded as the control. Δ*E* was obtained by comparing the results of color determination at each time point with the control. The result of this study was represented as Δ*E*, L*, a*, and b* plotted against time in min. Measurements were done in triplicate.

### In Vitro Delivery of FITC‐Dextran 150 kDa and Fluorescein Sodium

The in vitro transdermal delivery of FITC‐dextran 150 kDa and fluorescein sodium was evaluated with a Franz diffusion setup (Permergear, Hellertown, PA, USA). A 12 mL volume of prewarmed PBS (pH 7.4) was added to the receptor compartment as a releasing medium and was maintained at 37 ± 1 °C. The full‐thickness porcine skin was obtained as described previously and stored at −20 °C.^[^ ^51^ ^]^ Prior to testing, the skin was defrosted and equilibrated in PBS (pH 7.4) for 1 h. Using cyanoacrylate adhesive (Tex Year Industries Inc., Taibei, Taiwan) the skin was attached to the donor compartment with the SC facing up. The DMAP was inserted into the skin with a manual force applied for 30 s. A cylindrical metal weight was placed on the inserted DMAP to keep the patch in position. Then the donor compartment was mounted carefully to the receptor compartment without generating air bubbles and a piece of Parafilm M was used to wrap between the two compartments to minimize evaporation of the releasing medium and avoid contamination from the environment. A magnetic stir bar (4 mm × 10 mm) in the releasing medium was set at 600 rpm to homogenize the dissolution of compounds. A 200 µL sample was taken out from the sample arm of the receptor compartment at predetermined intervals and the same volume of fresh PBS was added subsequently into the receptor compartment after sampling. The sample was diluted to the linear range and analyzed with the Microplate Reader (FLUOstar Omega, Isogen Life Science, Netherlands) using the pre‐developed analytical method. The excitation wavelength was set to 485 nm and the emission wavelength was set to 520 nm for both FITC‐dextran 150 kDa and fluorescein sodium with a gain of 1000 and 700, respectively.

### Dye Retention Test within Silica

A 1 mg mL^−1^ stock solution of crystal violet was prepared by dissolving a defined amount of crystal violet in PBS (pH 7.4) and gredient diluting it to obtain the concentrations in the range of 0.124 to 4 µg mL^−1^. The absorbance of the solutions was scanned using an Agilent Cary 60 UV–vis spectrophotometer (Agilent Technologies UK Ltd., Stockport, UK). The calibration curve was constructed by plotting absorbance at the wavelength of 592 nm on the *Y*–axis and the nominal concentration on the *X*–axis.

The amount of dye dissolving from the silica was quantified. A 2 g of M50 was weighed and was suspended in 1 mL of PBS (pH 7.4) followed by vortexing for 30 s. The suspension was centrifuged at a speed of 5000 rpm for 10 min and the supernatant was analyzed with the UV–vis spectrophotometer.

The dye deposited in the skin after the dissolution of the needles of DMAP was evaluated. The baseplate was removed from the skin after 3 h and the skin was cut into pieces and homogenized with a TissueLyser LT (QIAGEN, Hilden, Germany) for 15 min at a speed of 50 Hz. The skin sample was centrifuged at a speed of 5000 rpm for 10 min and the absorbance of the supernatant was analyzed with the UV–vis spectrophotometer at the wavelength of 592 nm.

### Silica Residue Test after Insertion

The silica particle was covalently labeled with FITC in order to be able to trace the location of the silica and evaluate its potential migration out of the DMAP during insertion and dissolution. Briefly, FITC was reacted with APTES by mixing 1.27 mg of FITC with 2 µL of APTES dissolved in 200 µL of absolute ethanol and stirring this mixture at room temperature for 1 h. Then, this mixture was added to a suspension of the silica powder (100 mg in 1 mL of ethanol). After stirring at room temperature overnight, the labeled particles were collected by centrifugation and washed with ethanol before drying and storing at room temperature protected from light until further use. Successful labeling of the silica powder using FITC was confirmed by fluorescence microscopy of the silica powder using an EVOS FL fluorescence microscope (Thermo Fisher Scientific, Waltham, USA). The fluorescent labeling was further confirmed by measuring the fluorescence signal of serial gradient concentrations of an aqueous suspension of the labeled silica powder using a Microplate Reader (BMG LABTECH, Offenburg, Germany) with an excitation wavelength of 485 nm and an emission wavelength of 520 nm. A calibration curve was established by plotting the fluorescence signal with labeled silica fluorescence in an aqueous suspension so that the concentration of silica in the sample could be determined.

The FITC‐labeled silica was loaded onto the baseplate material and cast onto the blank DMAP. The DMAP was inserted into the skin and visualized with a multi‐photon scanning microscope using the method provided in “Morphology Observation” to check the distribution of the silica. The DMAP was removed from the skin after 3 h and the skin was visualized again to investigate the residual of silica after removal. The skin was cut into pieces and homogenized with TissueLyser for 15 min at a speed of 50 rpm. A 200 µL of the skin homogenate was taken and the fluorescence signal was analyzed by the plate reader with skin treated with unlabeled silica baseplate DMAP as a blank.

### Cytotoxicity Test

To confirm the effect of PLA+M50 (15:1) and PLA+M180 (20:1) on human dermal fibroblast cells (3T3L1 cells), cell viability was evaluated by 3‐(4,5‐dimethylthiazol‐2‐yl)−2,5‐diphenyl tetrazolium bromide (MTT), LIVE/DEAD and cell proliferation assays. Blank DMAPs were fabricated with no drug but PVP and PVA in the needle to form the needle shape and two baseplate formulations in the backing layer, B1 & B2. Human fibroblast cells were plated and incubated in DMAP samples cultured with complete DMEM culture medium for 72 h at 37 °C with 5% CO_2_. The MTT assay was conducted as previously described.^[^ ^52^ ^]^ Briefly, the culture medium was replaced with MTT solution (0.5 mg mL^−1^) in DMEM. After 5 h, the supernatant was removed, DMSO was added (200 µL), and the sample was gently shaken for 15 min to allow the dissolution of the formazan crystals. Optical absorbance was measured using a microplate reader Synergy H1 microplate reader (Biotek) at 570 nm. Cell culture plate seeded was used as the positive control, and Triton X‐100 (1%) was used as a negative control. To confirm cell viability, LIVE/DEAD staining was conducted. First, DMAP samples were added to fibroblast cell culture for 72 h. The cells were then stained for 5 min at room temperature in 5 µg mL^−1^ calcein AM and 5 µg mL^−1^ ethidium homodimer‐1 (Molecular Probes, Eugene, Oregon). In addition, cell proliferation was also evaluated via DNA content assay. The amount of DNA in the cells attached to DMAP samples was determined using Quant‐iT PicoGreen dsDNA Reagent and Kits (Molecular Probes, Life Technologies Corp) based on the manufacturer's instructions. The samples were rinsed with PBS (pH 7.4) three times and submerged in 1 mL of lysis buffer containing 10 mM Tris (pH 8), 1 mM EDTA, and 0.2% v/v Triton X‐100. To promote DNA release, the samples were vortexed for 10 s every 5 min for a total of 30 min and were kept on ice throughout the entire process. The samples were thawed on ice and homogenized for 10–15 min. The samples were subsequently mixed with 100 µL of DNA‐binding fluorescent dye solution, and the fluorescence intensity was measured at an excitation wavelength of 480 nm and an emission wavelength of 520 nm. Lambda DNA was used for the standard curve to calculate the amount of DNA. All sample analyses were conducted in triplicate.`

## Conflict of Interest

The authors declare no conflict of interest.

## Supporting information

Supporting Information

## Data Availability

The data that support the findings of this study are available from the corresponding author upon reasonable request.
