# Supplementary material for: Design of a Novel Delivery Efficiency Feedback System for Biphasic Dissolving Microarray Patches Based on Poly(Lactic Acid) and Moisture‐Indicating Silica
Source: Adv Healthc Mater. 2024 Mar 19;13(17):2304082. doi: 10.1002/adhm.202304082 (PMC11468354; doi:10.1002/adhm.202304082)
Supplement: Supplementary file 1 — Supporting Information [file ADHM-13-2304082-s001.pdf]

ADVANCED HEALTHCARE MATERIALS

Supporting Information

for *Adv. Healthcare Mater.*, DOI 10.1002/adhm.202304082

Design of a Novel Delivery Efficiency Feedback System for Biphasic Dissolving Microarray Patches Based on Poly(Lactic Acid) and Moisture-Indicating Silica

*Huanhuan Li, Qonita Kurnia Anjani, Aaron R. J. Hutton, Juan Luis Paris, Natalia Moreno-Castellanos, Achmad Himawan, Eneko Larrañeta and Ryan F. Donnelly**

Design of a novel delivery efficiency feedback system for biphasic dissolving microarray patches based on poly(lactic acid) and moisture-indicating silica

Huanhuan Li[1], Qonita Kurnia Anjani[1], Aaron R.J. Hutton[1], Juan Luis Paris [1,2], Natalia Moreno-Castellanos[3], Achmad Himawan[1,4], Eneko Larrañeta[1], Ryan F. Donnelly[1]*

*Corresponding author (Ryan F. Donnelly) 97 Lisburn Road, Belfast BT9 7BL, Northern Ireland, UK, r.donnelly@qub.ac.uk

1. School of Pharmacy, Queen's University Belfast, BT9 7BL, United Kingdom
2. Instituto de Investigación Biomédica de Málaga y Plataforma en Nanomedicina-IBIMA Plataforma BIONAND, Málaga, Spain.
3. Basic Science Department, Faculty of Health, Universidad Industrial de Santander, Bucaramanga 680001, Colombia
4. Department of Pharmaceutical Science and Technology, Faculty of Pharmacy, Universitas Hasanuddin, Makassar 90245, Indonesia

Supporting Information

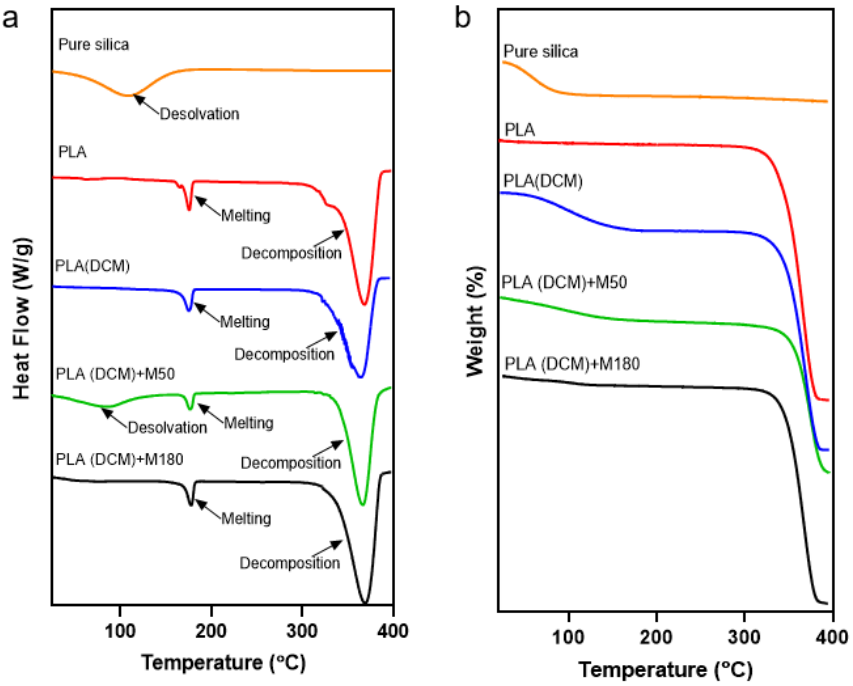

Figure S1. DSC (a) and TGA (b) thermograms for pure silica, pure PLA, PLA dissolved in DCM and the two baseplate films, PLA:M50 (15:1) and PLA:M180 (20:1).

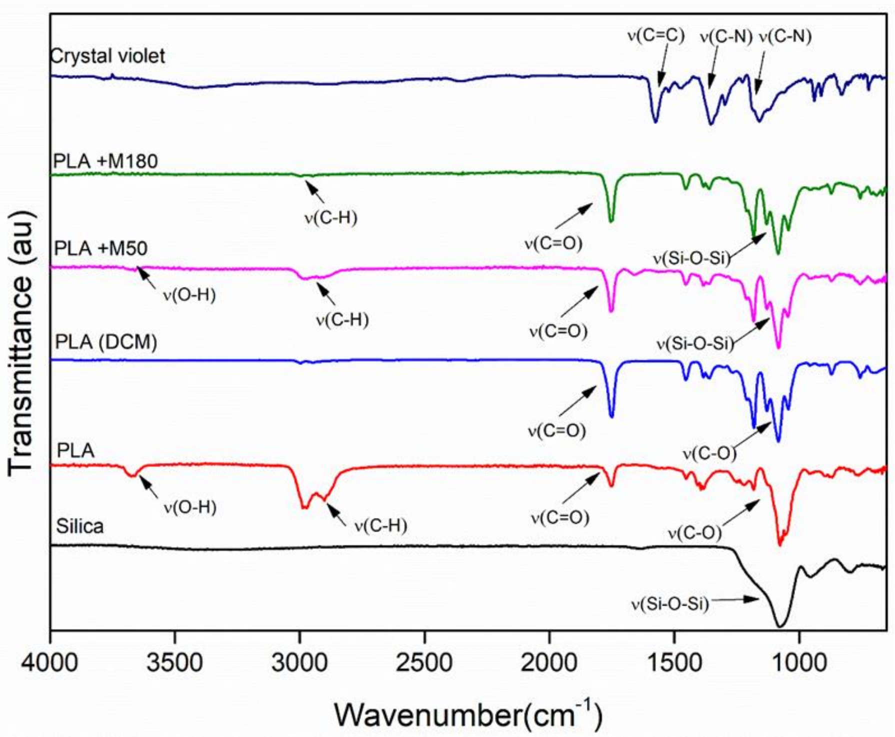

Figure S2. FTIR transmittance spectra of pure silica, pure PLA, PLA dissolved in DCM, the two baseplate films, PLA:M50 (15:1) and PLA:M180 (20:1), and crystal violet dye.

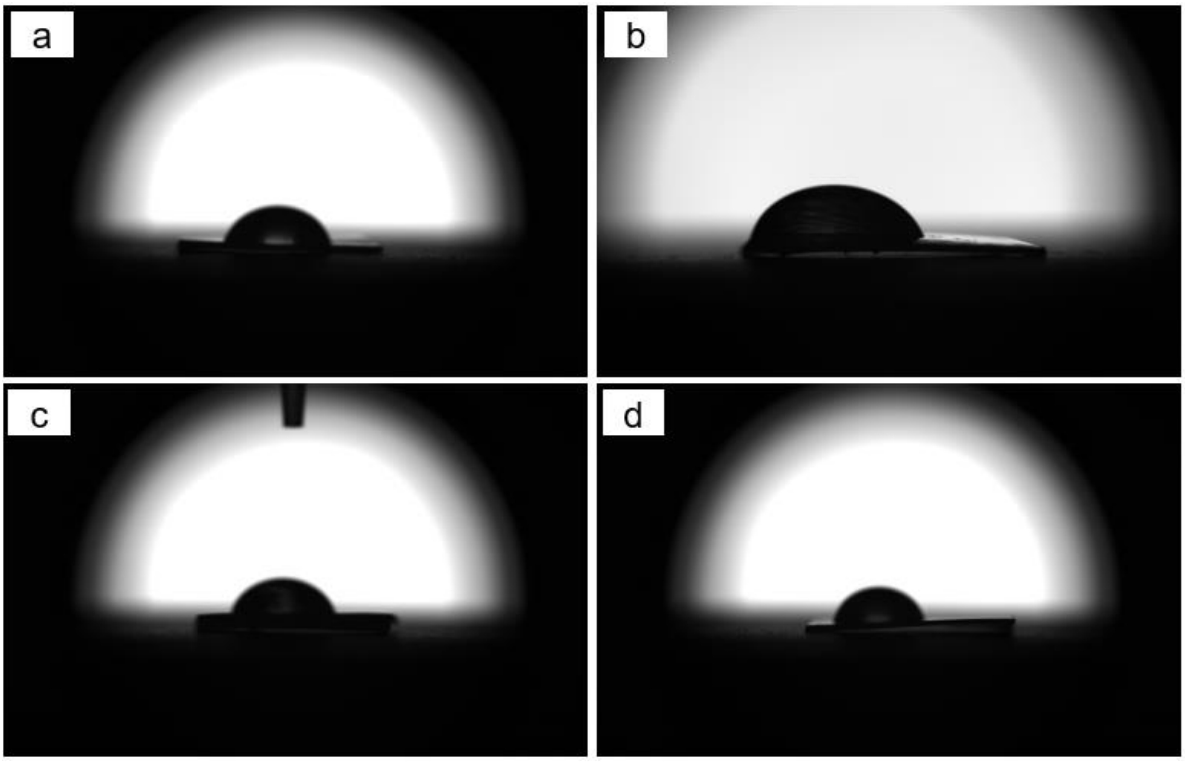

Figure S3. Static water contact angle on PLA-based film(a), PVP-based film (b), PLA+M180 (20:1) (c) and PLA+M50 (15:1) film (d).

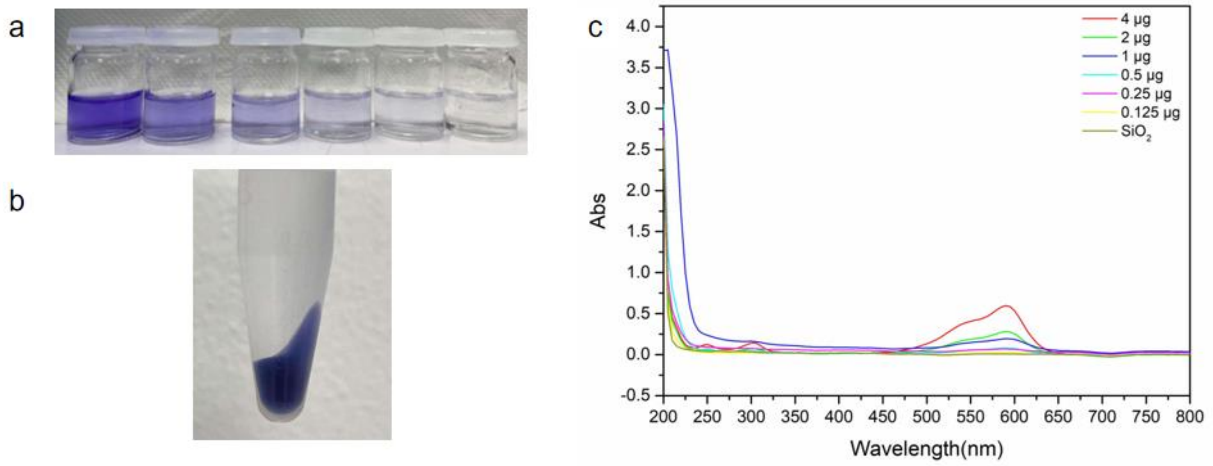

Figure S4. Images of crystal violet solution with gradient concentration (a), silica suspension in PBS after centrifugation (b), UV scan of gradient solutions and supernatant from b (c).
